# Droplet interface bilayer reconstitution and activity measurement of the mechanosensitive channel of large conductance from *Escherichia coli*

**DOI:** 10.1098/rsif.2014.0404

**Published:** 2014-09-06

**Authors:** Hanna M. G. Barriga, Paula Booth, Stuart Haylock, Richard Bazin, Richard H. Templer, Oscar Ces

**Affiliations:** 1 Membrane Biophysics Platform, Institute of Chemical Biology and Department of Chemistry, Imperial College London, South Kensington, London SW7 2AX, UK; 2 Department of Biochemistry, University of Bristol, Bristol BS8 1TD, UK; 3 Pfizer Global Research and Development, Sandwich CT13 9NJ, UK

**Keywords:** droplet interface bilayers, mechanosensitive channel of large conductance, 1,2-dioleoyl-*sn*-glycero-3-phosphocholine, fluorescence

## Abstract

Droplet interface bilayers (DIBs) provide an exciting new platform for the study of membrane proteins in stable bilayers of controlled composition. To date, the successful reconstitution and activity measurement of membrane proteins in DIBs has relied on the use of the synthetic lipid 1,2-diphytanoyl-*sn*-glycero-3-phosphocholine (DPhPC). We report the functional reconstitution of the mechanosensitive channel of large conductance (MscL) into DIBs composed of 1,2-dioleoyl-*sn*-glycero-3-phosphocholine (DOPC), a lipid of significantly greater biological relevance than DPhPC. MscL functionality has been demonstrated using a fluorescence-based assay, showing that dye flow occurs across the DIB when MscL is gated by the cysteine reactive chemical 2-(trimethylammonium)ethyl methane thiosulfonate bromide (MTSET). MscL has already been the subject of a number of studies investigating its interaction with the membrane. We propose that this method will pave the way for future MscL studies looking in detail at the effects of controlled composition or membrane asymmetry on MscL activity using biologically relevant lipids and will also be applicable to other lipid–protein systems, paving the way for the study of membrane proteins in DIBs with biologically relevant lipids.

## 1. Introduction

Droplet interface bilayers (DIBs) provide an ideal method to miniaturize planar bilayer studies. Their applications range from ultra-stable bilayers for single-channel recordings, droplet networks and arrays to the study of transport across membranes [1–3]. DIBs exploit the principle that an aqueous droplet submerged in an oil–lipid mixture will spontaneously acquire a lipid monolayer at the water–oil interface. When two such droplets are brought together, the oil between the two droplets is displaced and a stable bilayer forms, which can last weeks [1]. Another exciting prospect for DIBs is the ability to reconstitute proteins across the DIB, in particular channels and to characterize channel behaviour and the effects of membranes. Unlike systems which use patch clamps, DIBs provide an environment where the membrane is not distorted by applied pressures. A number of proteins including the potassium channel Kcv and the light-driven proton pump bacteriorhodopsin have already been documented in DIB studies and the list is continually expanding [1]. The reconstitution conditions for each protein vary, ranging from a pH gradient between droplets to applying a voltage difference, but conductance across the DIB has successfully been measured in all of the cases [4].

To date, the majority of studies have used the lipid 1,2-diphytanoyl-*sn*-glycero-3-phosphocholine (DPhPC) to form stable DIBs and reconstitute some of the more stable membrane proteins such as staphylococcal α-haemolysin (αHL) [5] and anthrax toxin pores [6]. The reconstitution of eukaryotic ion channels has also been reported but again using the synthetic lipid DPhPC [1]. There have however been few that report the use of other lipids in DIBs. One example is the use of mixtures of photopolymerizable and non-polymerizable lipids to produce photoactivated cross-linking for phototriggered drug delivery systems [7]. To the best of our knowledge, there has not yet been reported the reconstitution of membrane proteins into DIBs formed from biologically relevant lipids. Here, we present evidence for the functional reconstitution and activity measurement of the mechanosensitive channel of large conductance (MscL) from *Escherichia coli* into DIBs composed of 1,2-dioleoyl-*sn*-glycero-3-phosphocholine (DOPC). Activity measurements used a fluorescence-based assay [8], removing the need for probes which disrupt the membrane such as those used during patch clamp experiments. This work demonstrates the use of DIBs to create more biologically applicable systems, in terms of both the lipid composition and the sensitivity of the proteins studied.

The MscL from *E. coli* is a pentameric integral membrane protein and forms a large non-selective channel for osmolites. It has one of the largest conformational changes known in membrane proteins between its closed and open states and has a pore size of 30–40 Å [9]. Each monomer is predominantly α-helical and contains two transmembrane domains, cytoplasmic N and C terminal domains and a central periplasmic domain [10].

Integral membrane proteins are known to interact dynamically with the membrane, the precise mechanism of which has been the focus of a number of studies. A large number of these have focused on some of the more widely studied proteins bacteriorhodopsin and diacylglycerol kinase and investigated the influence of lipid composition, charged headgroups, interfacial properties, the hydrophobic effects and mechanical forces within the bilayer [11–15].

Although the precise membrane deformations which trigger MscL gating are still unclear, MscL responds to mechanical forces within the bilayer for which the lipid–protein interactions form an essential role. There are a number of MscL studies which look at the effects of lipids on MscL such as the lipid chain length, direct interactions and anionic phospholipids [8,16]; however, to the best of our knowledge, there has been no report of the functional reconstitution of MscL into DIBs.

**Figure 1.**
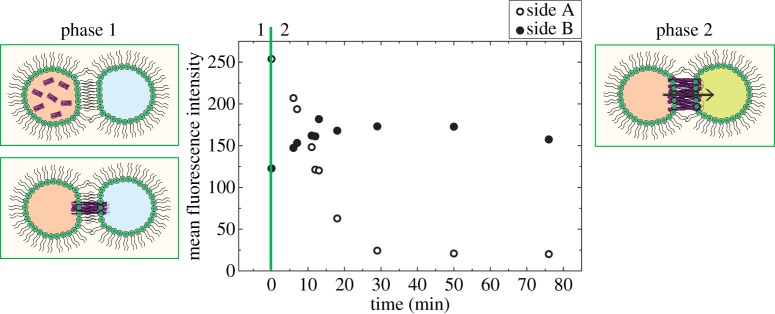
Plot showing fluorescence changes in a DIB in the presence of both MscL and MTSET. Side A is the MscL- and calcein-containing droplet, which decreases in intensity, whereas side B, which contains sucrose and the gating chemical MTSET, shows a fluorescence increase as calcein enters the droplet through the MscL channel. This process has also been shown schematically, starting with phase 1 where the DIB forms and MscL reconstitutes, leading to phase 2 where MscL is gated by the MTSET in the other droplet, opens and a fluorescence change can be seen as calcein flows across the DIB. (Online version in colour.)

In this report, we demonstrate the functional reconstitution of MscL into DOPC DIBs followed by subsequent MscL activation via a cysteine-reactive compound, 2-(trimethylammonium)ethyl methane thiosulfonate bromide (MTSET). Purified MscL is reconstituted into DOPC extruded vesicles in the presence of the self-quenching fluorescent dye calcein. A droplet of this calcein solution is placed into a hexadecane environment, enabling the formation of a monolayer at the interface. If this droplet is then brought into contact with another non-fluorescent DOPC droplet, the purified MscL will reconstitute into the bilayer that forms when the two droplets form a DIB. This has been shown schematically in figure 1, phase 1. If the non-fluorescent droplet also contains the gating chemical MTSET but no calcein, then the reconstituted MscL will open, enabling dye flow to occur from one droplet to the other as demonstrated in figure 1, phase 2. We propose that the reason for this increase is the movement of calcein from the droplet in which it is quenched to the droplet containing no dye, upon which a fluorescence increase is seen as the dye unquenches in solution. We present data showing that this dye transfer only occurs in the presence of activated MscL, demonstrating the functional reconstitution of MscL into a DIB.

## 2. Experimental procedures

### 2.1. Mechanosensitive channel of large conductance expression and purification

Glycerol stocks of the MscL expression clone were a gift from Paula Booth. The *E. coli* BL21 (DE3) cells carrying the kanamycin-resistant pET28a vector were expressed and purified using a modified protocol of Blount *et al*. [17]. A mutant form of MscL (G22C F93W) was expressed which is known to react with MTSET [18]. All the materials were purchased from Sigma Aldrich apart from the protease inhibitor, which was purchased from Roche.

### 2.2. Lipid vesicle preparation

DOPC was purchased from Avanti Polar Lipids. Lipids for MscL reconstitution were hydrated at 6.7 mM in 20 mM HEPES, 100 mM KCl, 40 mM *n*-octyl-β-d-glucopyranoside (OG) with either 50 mM (controls) or 0.5 mM (assay) calcein and freeze–thawed four times. Lipids for the MscL activation droplet were hydrated in 20 mM HEPES, 100 mM KCl and 0.5 M sucrose. Lipids were extruded through a 100 nm polycarbonate filter using an Avanti Mini Extruder to form mixed lipid–detergent small unilamellar vesicles (SUVs). These were monitored by dynamic light scattering both pre- and post-MscL reconstitution, confirming their stability throughout.

### 2.3. Mechanosensitive channel of large conductance reconstitution

Purified MscL was added at a lipid : protein molar ratio of 1000 : 1 to the SUV sample containing calcein. Afterwards 150 mg of BioBeads was added and the sample was left overnight on rollers at 4°C.

### 2.4. Droplet interface bilayer assay

A droplet of reconstituted MscL was injected into an enclosed polydimethylsiloxane channel filled with hexadecane. Immediately after, a droplet of the extruded vesicles in buffer but without the calcein were mixed with 300 mM MTSET and injected into the hexadecane channel. An excess of MTSET was used to ensure that MTSET did not limit the fluorescence changes. Both droplets were left to equilibrate to allow monolayer formation. Subsequently, the two droplets were brought together by creating a flow of oil within the channel and the change in fluorescence of each droplet measured using a fluorescence inverted microscope (Nikon Eclipse TE 2000-E) with a mercury lamp (Olympus) and a QICAM camera (QImaging, Media Cybernetics UK). Fluorescence images were obtained using an fluorescein isothiocyanate dichroic filter (460–490 nm excitation; Chroma Technology Corp). Mean fluorescence intensity for each droplet was calculated via the integration of the droplet intensity using ImageJ.

## 3. Results

### 3.1. Droplet interface bilayer assay controls

Controls using 50 mM calcein were undertaken to ensure that any changes in fluorescence seen were solely due to calcein flow through the activated MscL channel. The high calcein concentration compared with the assay (0.5 mM) enabled the sensitive detection of minute changes in fluorescence.

**Figure 2.**
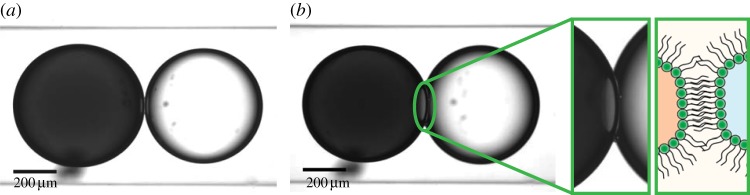
Demonstration of DOPC DIB stability over a time period of 1 h at (*a*) *t* = 0, (*b*) *t* = 1 h, where the dark droplet is calcein loaded (50 mM) and the lighter droplet sucrose loaded. A close-up of the stable DIB and a schematic has also been added to (*b*) to demonstrate DIB formation. (Online version in colour.)

The stability of the DOPC DIB in the presence of buffers and a dye gradient was demonstrated and shown to be stable for an hour without the two droplets merging, as shown in figure 2.

**Figure 3.**
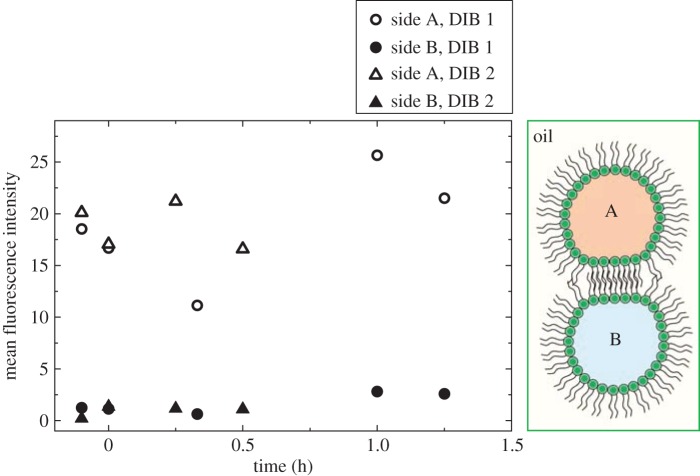
Control plot showing fluorescence changes in the DIB droplets where DIB 1 contains MTSET but no MscL and DIB 2 contains MscL but no MTSET. Neither DIB shows any fluorescence change in side A or B, where side A is calcein loaded (0.5 mM) and side B is sucrose loaded. (Online version in colour.)

To prove that the gating chemical MTSET and/or reconstituted MscL did not cause changes in fluorescence or movement of dye across the DIB unless MscL was gated, the fluorescence of a DIB was monitored, firstly, in the absence of MscL, but with MTSET and, secondly, in the presence of reconstituted MscL, but absence of MTSET. No fluorescence changes were seen in either case as shown in figure 3.

### 3.2. Measuring mechanosensitive channel of large conductance activity

After successful controls, we demonstrate the measurement of MscL activity in DIBs. This is shown in figure 1, where over a time period of 80 min after DIB formation the mean fluorescence intensity in one droplet decreases while it increases in the other. This dye flow only occurs in the presence of MscL and MTSET, hence we can conclude the successful reconstitution of MscL into a DIB and measurement via a fluorescence-based assay.

## 4. Discussion

Here we show the successful transfer of purified MscL into DIBs and demonstrate its functionality using a fluorescence-based assay. We show that dye flow only occurs upon MscL gating, implying that calcein flow and hence fluorescence changes are only measured when MscL is active and calcein can diffuse through the pore and down the concentration gradient.

While figure 1 clearly shows that MscL is active, we note that the fluorescence does not start from zero and attribute this to the time delay of a few minutes between DIB formation and measurement commencing, which occurs in both the control and final measurement. Our controls show that at *t* = 0 there is zero fluorescence, therefore all changes in fluorescence are significant and due solely to MscL activity. Additionally, the final fluorescence of sides A and B are not equal. MTSET is a powerful gating compound for MscL [9] and has a smaller molecular weight than calcein. This implies that, upon MscL gating, excess MTSET will be free to diffuse through MscL into side A. During the monolayer formation, it is likely that MscL will be present in excess vesicles in side A, at the lipid–oil interface and in the final DIB as the vesicles migrate to the water–oil interface [1]. The diffusion of MTSET into side A would activate any MscL present in the monolayer, causing further dye leakage from side A into the surrounding oil medium. This would be undetectable owing to the low concentrations of any dye present in the oil but could account for the marked fluorescence reduction in side A in comparison with the increase in side B in the DIB.

## 5. Conclusion

In conclusion, we show successful functional reconstitution of MscL into a DIB composed of DOPC. This has been demonstrated using a fluorescence assay whereupon dye flow occurs only upon MscL gating. This will make a significant contribution to future studies on MscL and membrane asymmetry as well as applications to droplet networks and membrane proteins in biologically relevant lipids such as those previously reported in the literature.
